# Supplementary material for: Tead1a Initiates Transcriptional Priming Through the TEAD1a/YAP‐Notch1‐Spi1/Cebpα Axis to Promote Neutrophil Fate
Source: Adv Sci (Weinh). 2025 Aug 28;12(41):e05441. doi: 10.1002/advs.202505441 (PMC12591127; doi:10.1002/advs.202505441)
Supplement: Supplementary file 4 — Supplementary Dataset [file ADVS-12-e05441-s004.zip › 3-1.zip!/3-1/02-1.cluster/cluster1/Enrich/ego_BP.pdf]

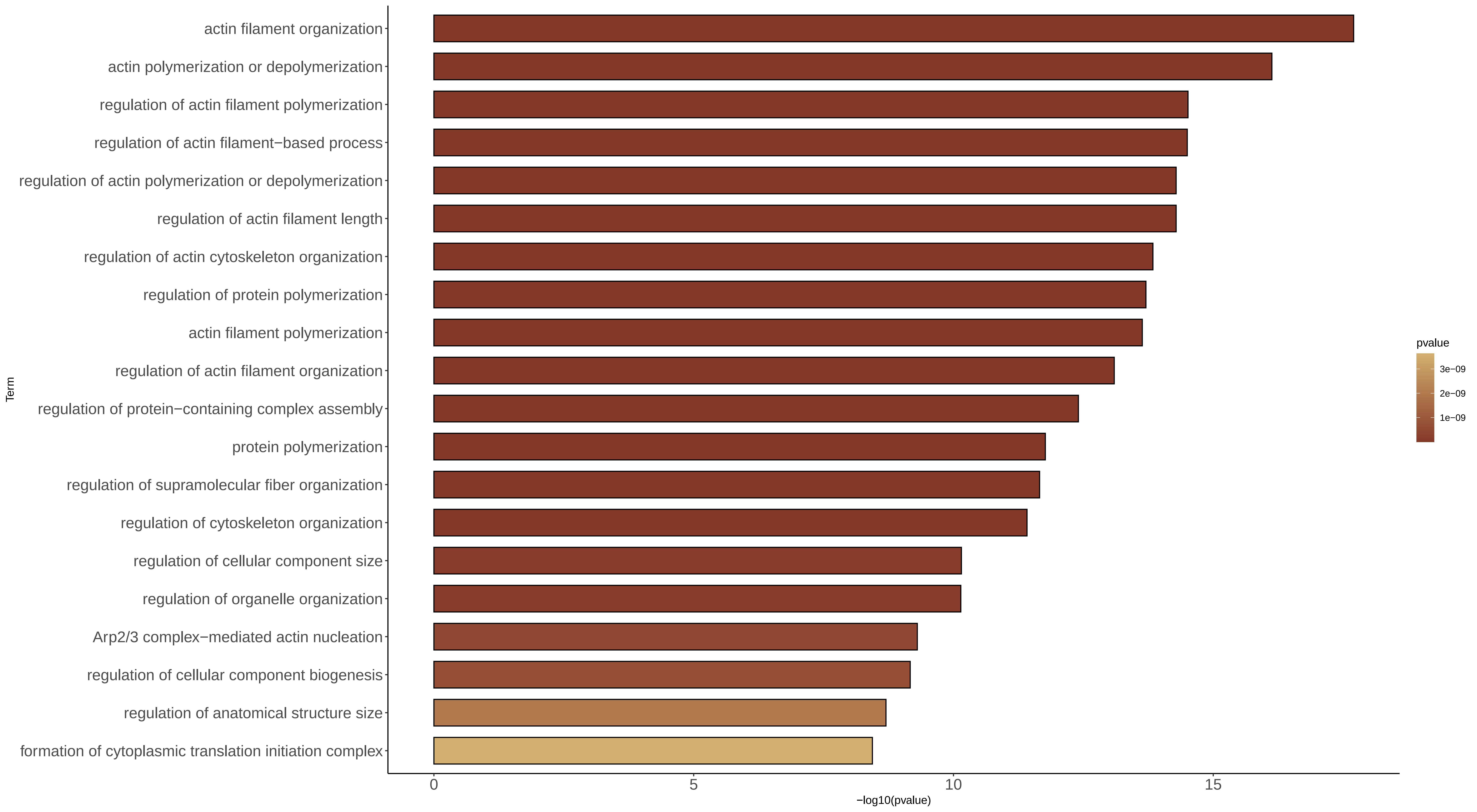
actin filament organization
actin polymerization or depolymerization
regulation of actin filament polymerization
regulation of actin filament−based process
regulation of actin polymerization or depolymerization
regulation of actin filament length
regulation of actin cytoskeleton organization
regulation of protein polymerization
actin filament polymerization
regulation of actin filament organization
regulation of protein−containing complex assembly
protein polymerization
regulation of supramolecular fiber organization
regulation of cytoskeleton organization
regulation of cellular component size
regulation of organelle organization
Arp2/3 complex−mediated actin nucleation
regulation of cellular component biogenesis
regulation of anatomical structure size
formation of cytoplasmic translation initiation complex
Term
0
5
10
15
−log10(pvalue)
pvalue
3e−09
2e−09
1e−09
